# Mechanobiology of the diabetic cardiomyocyte: insulin signaling, titin elasticity, and multiscale mechanical dysfunction

**DOI:** 10.1007/s12551-026-01444-9

**Published:** 2026-06-10

**Authors:** Mustafa Kaçmaz, Evelyn Halupka, Innas Sultana, Simin Delalat, Ibrahim El-Battrawy, Muchtiar Khan, Loek van Heerebek, Nazha Hamdani

**Affiliations:** 1 Medical Faculty, Institute of Physiology, Department of Cellular and Translational Physiology, Ruhr-University Bochum, Bochum, Germany; 2 Medizinische Klinik II, Kardiologie, Klinikum der Ruhr-Universität Bochum, Bochum, Germany; 3 Department of Cardiology, OLVG, Amsterdam, The Netherlands; 4 HCEMM-SU, Cardiovascular Comorbidities Research Group, Center for Pharmacology and Drug Research & Development, Department of Pharmacology and Pharmacotherapy, Semmelweis University, Budapest, Hungary; 5 Department of Physiology, Cardiovascular Research Institute Maastricht, Maastricht University, Maastricht, The Netherlands

**Keywords:** Cardiomyocytes, Electromechanical processes, Diabetes mellitus

## Abstract

Cardiomyocyte function emerges from tightly coupled electromechanical processes that span sarcomeric force generation, titin-based elasticity, excitation–contraction coupling, mitochondrial ATP supply, and mechanotransductive adaptation to load. Insulin signaling integrates these processes across molecular, cellular, and organ scales, thereby contributing to cardiomyocyte mechanical homeostasis. In cardiomyocytes, canonical insulin signaling is initiated by insulin receptor (IR) activation, recruitment of IRS-1/IRS-2, and downstream PI3K–Akt signaling. Through Akt-dependent modulation of mTOR, GSK-3β, and FOXO transcription factors, insulin aligns energy availability with mechanical demand while supporting structural integrity of sarcomeres, Z-disc/costameric networks, and intercalated disc architecture. Disruption of insulin signaling and insulin resistance in type 2 diabetes (T2DM) remodel cardiomyocyte mechanics by altering myofilament calcium sensitivity, shifting contractile protein expression, perturbing titin isoform composition and phosphorylation, impairing calcium cycling and β-adrenergic microdomain signaling, and inducing mitochondrial dysfunction with oxidative stress (Fig. 1). These changes manifest as altered force–pCa relations, reduced contractile reserve, prolonged relaxation, increased passive stiffness, and modified viscoelastic behavior, as measured by quantitative biophysical assays (Fig. 2). Here, we synthesize mechanistic pathways linking insulin signaling to cardiac mechanics; summarize evidence for T2DM-induced cardiomyocyte dysfunction across species and disease stages; describe mechanotransduction failure in diabetes involving costameres, integrins/FAK, and stretch-responsive pathways such as YAP/TAZ; and provide an overview of quantitative tools to measure cardiomyocyte mechanics including AFM, TFM, nanoindentation, optical/magnetic tweezers, skinned-cell mechanics, real-time calcium–contractility platforms, engineered heart tissues, and microphysiological heart-on-chip systems. Finally, we discuss therapeutic perspectives with emphasis on interventions that restore mechanical homeostasis through titin phosphorylation, reduction of AGE-driven stiffening, and normalization of oxidative and inflammatory stress, including SGLT2 inhibitors and GLP-1 agonists.

## Introduction: Linking diabetes, insulin, and cardiac mechanics

**Fig. 1:**
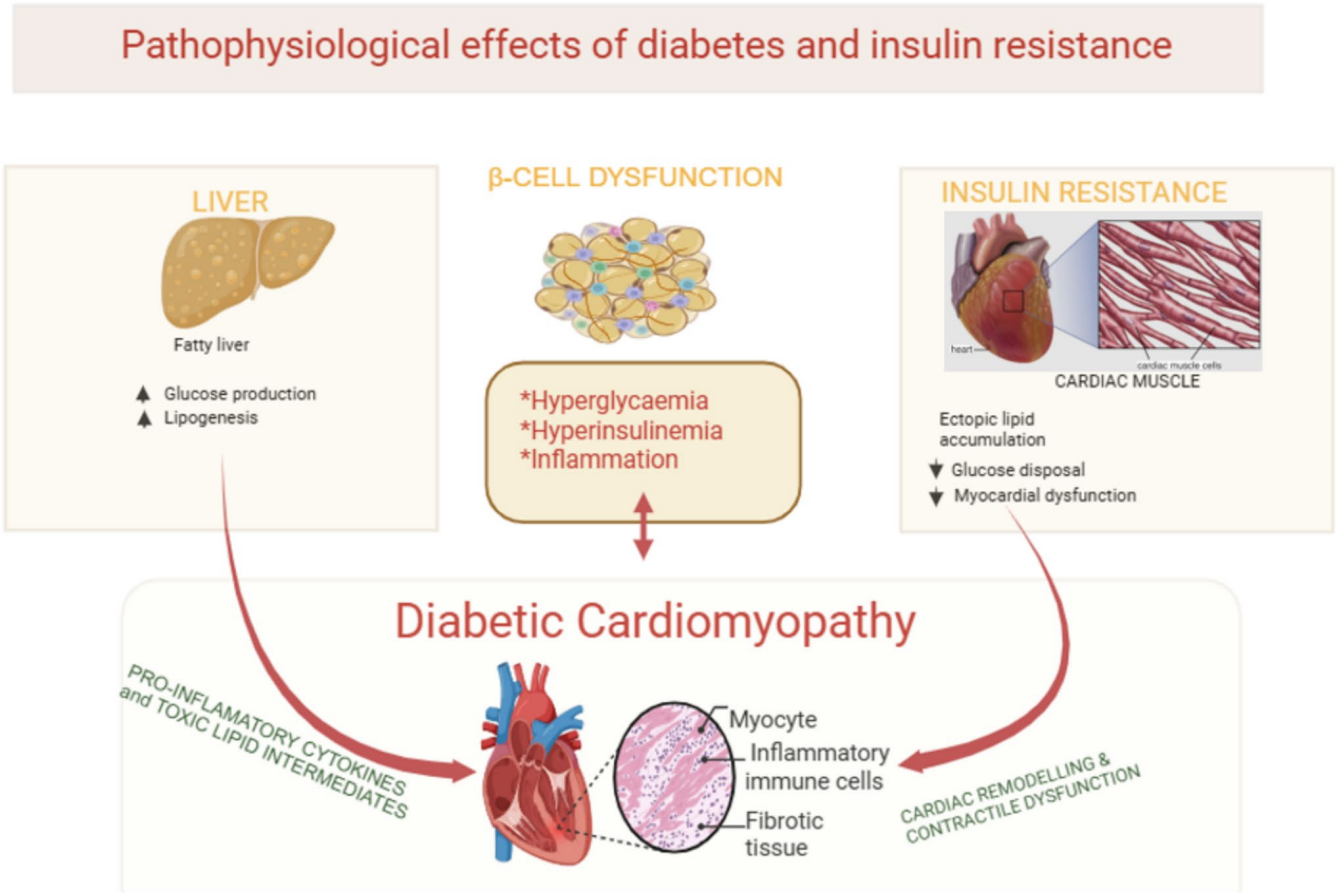
Pathophysiological interplay between β-cell dysfunction, insulin resistance, and the development of diabetic cardiomyopathy. The figure illustrates how β-cell dysfunction and insulin resistance lead to hyperglycemia, hyperinsulinemia, and inflammation. Metabolic disturbances in the liver and skeletal muscle promote lipid accumulation and impaired glucose handling. These systemic alterations converge on the heart, causing inflammation, fibrosis, cardiomyocyte dysfunction, and cardiac remodeling, ultimately resulting in diabetic cardiomyopathy

Diabetes mellitus (DM) is strongly linked to heart failure development and progression, and diabetic cardiomyopathy is increasingly recognized as a disorder of coupled metabolism and mechanics rather than metabolism alone. Because DM, particularly type 2 diabetes mellitus (T2DM), enriches HFpEF phenotypes yet also accelerates ischemic injury and HFrEF progression, the mechanical pathways discussed here are likely to contribute across HF subtypes through shared mechanisms (stiffness, energetic inefficiency) and divergent drivers (ischemia-associated remodeling) (Li et al. 2024; Mgbemena et al. 2021). Cardiomyocytes are electromechanical structures that continuously sense and respond to their mechanical microenvironment, including extracellular matrix (ECM) stiffness, load, stretch, shear-associated cues, and cell–cell coupling forces. Mechanical performance depends on coordinated regulation of sarcomeric cross-bridge cycling, calcium handling kinetics, titin-based elasticity, cytoskeletal architecture, mitochondrial ATP production, and mechanotransductive feedback loops that adapt intracellular, extracellular, transcriptional, and post-translational programs to workload. Insulin is central to this coordination because cardiomyocytes express abundant insulin receptors and insulin signaling interfaces with contractility, relaxation, and structural maintenance via PI3K/Akt and interacting kinase networks (Abel 2021; Pantazi et al. 2022; Caturano et al. 2024) (Fig. 1). In T2DM and insulin resistance, these coupling mechanisms become dysregulated, leading to impaired excitation–contraction coupling, altered myofilament properties, increased passive stiffness, and maladaptive remodeling. A quantitative mechanistic perspective is needed because the phenotype ultimately presents as measurable changes in force production, stiffness, viscoelasticity, and energetic efficiency. Key outputs discussed here include active tension (T_active_), passive tension (F_passive_), myofilament Ca^2^⁺ sensitivity (pCa_50_), cooperativity (nHill), rate of force redevelopment (ktr), and stiffness/viscoelastic parameters derived from indentation-based methods (Gonçalves-Rodrigues et al. 2020).

## Insulin signaling pathways underlying cardiomyocyte mechanical homeostasis

Insulin signaling plays a pivotal role in cardiomyocyte mechanical homeostasis by coordinating contractile protein expression, calcium cycling, sarcomeric elasticity, mitochondrial energetics, and substrate utilization. In cardiomyocytes, canonical insulin signaling is initiated by ligand binding to the insulin receptor (IR), which triggers receptor autophosphorylation and the recruitment of insulin receptor substrates IRS-1 and IRS-2. This activates phosphoinositide 3-kinase (PI3K) and protein kinase B (Akt), a central signaling hub mediating insulin’s intracellular effects. Downstream, Akt modulates mammalian target of rapamycin (mTOR), glycogen synthase kinase-3β (GSK-3β), and forkhead box O (FOXO) transcription factors (De Meyts et al. 2016; Achter et al. 2024). Through this signaling network, insulin aligns energy supply with mechanical demand and preserves the structural integrity of the contractile apparatus.

At the myofilament level, insulin signaling regulates myosin heavy chain (MHC) isoform composition, which directly determines cross-bridge kinetics and power output. Cardiomyocyte-specific insulin receptor knockout (CIRKO) mice exhibit persistent expression of the fetal β-MHC isoform at markedly elevated levels (Belke et al. 2002). Compared with α-MHC, β-MHC displays lower actin-activated ATPase activity and slower cross-bridge cycling rates, resulting in reduced shortening velocity and diminished maximal mechanical power despite greater force per cross-bridge and improved energetic economy (Pope et al. 1980). This isoform shift, therefore, biases the myocardium toward a slower, energetically conservative contractile phenotype. Beyond isoform regulation, insulin signaling is required for physiological cardiac growth and maintenance of cardiomyocyte size (Belke et al. 2002). Combined deletion of IRS1/IRS2 (iCIRS12KO) or IR/IGF1R-DKO (iCI2RKO) leads to rapid transcriptional remodeling characterized by reduced serum response factor (SRF) activity and impaired expression of structural sarcomeric genes. Among these, disruption of myocardin, an SRF coactivator, and downregulation of PDZ-LIM domain protein 5 (Pdlim5), a Z-disc-associated cytoskeletal component, were detected (Riehle et al. 2022). Furthermore, ultrastructural analyses demonstrate early disorganization of sarcomeres and intercalated discs following loss of IR/IGF1R or IRS1/2 signaling (Riehle et al. 2022), establishing insulin pathways as essential guardians of structural homeostasis rather than purely metabolic regulators. At the organ level, CIRKO hearts exhibit reduced cardiac output and mechanical power (Belke et al. 2002), while broader disruption of IR/IGF1R or IRS1/2 signaling progresses to dilated cardiomyopathy with ventricular dilation and systolic failure (Riehle et al. 2022 underscoring the graded dependence of myocardial performance on intact insulin signaling.

Titin, the principal determinant of passive stiffness and diastolic compliance, represents another major biomechanical effector of insulin action. Insulin modulates titin mechanics through isoform composition and post-translational phosphorylation (Krüger et al. 2010a; Zhu et al. 2016). Chronic insulin exposure shifts titin expression toward the stiffer N2B isoform relative to the more compliant N2BA variant via PI3K/Akt- and mTOR-dependent mechanisms (PMID: 20184888). This effect is mediated through upregulation of the splicing factor RBM20, which promotes exon inclusion, favoring N2B titin production (Krüger et al. 2010b). Inhibition of PI3K or mTOR reduces RBM20 expression and attenuates N2B generation, demonstrating that titin alternative splicing is directly controlled by insulin signaling (Zhu et al. 2016) . In addition, insulin regulates titin stiffness by phosphorylating the N2-Bus and PEVK regions. Activation of PI3K/Akt/eNOS signaling enhances PKG-dependent phosphorylation at residues including S4099 and S4010, with a potential contribution from ERK1/2 pathways (Hopf et al. 2018). Insulin exposure increases phosphorylation levels of both titin isoforms in developing cardiomyocytes (Krüger et al. 2010a), thereby dynamically modulating passive tension and diastolic mechanics. Through coordinated control of titin splicing and phosphorylation, insulin fine-tunes myocardial compliance in response to metabolic state. The mechanical consequence depends on the modified titin region and the dominant kinase context.

Mechanical performance critically depends on excitation–contraction coupling, and insulin exerts direct positive inotropic effects (von Lewinski et al. 2005; Hsu et al. 2006) that extend beyond metabolic modulation. In failing human myocardium, insulin increases twitch force, enhances intracellular Ca^2^⁺ transients, and increases sarcoplasmic reticulum (SR) Ca^2^⁺ content (von Lewinski et al. 2005). These effects are mediated through PI3K-dependent pathways and, in part, reverse-mode Na⁺/Ca^2^⁺ exchange; they are independent from DAG or PKC activation and involve both Ca^2^⁺-dependent and Ca^2^⁺-independent mechanisms (H von Lewinski et al. 2005). Furthermore, insulin increases L-type Ca^2^⁺ current density (Hsu et al. 2006), indicating enhanced transsarcolemmal Ca^2^⁺ influx through voltage-gated channels.

At the transcriptional level, insulin also directly regulates Ca^2^⁺ handling proteins. In isolated cardiac myocytes and engineered heart tissue, insulin increases SERCA2a mRNA expression in a concentration-dependent manner via PI3K/Akt signaling and elevates the SERCA2a/phospholamban (PLB) ratio, thereby enhancing SR Ca^2^⁺ uptake capacity and accelerating relaxation kinetics (Fredersdorf et al. 2012). Insulin treatment can also reverse the decrease in SERCA2a activity observed in streptozotocin-induced diabetic models, indicating a direct stimulatory effect on Ca^2^⁺ reuptake machinery (Zhong et al. 2001).

Mitochondrial energetics represent the energetic backbone of contractile function, and insulin signaling directly coordinates mitochondrial dynamics and oxidative metabolism. Insulin promotes mitochondrial fusion by increasing expression of optic atrophy protein 1 (Opa1) and mitofusin 2 (Mfn2), enhancing membrane potential, oxygen consumption, and ATP production. These effects depend on Akt–mTOR–NFκB signaling. Silencing of Opa1 or Mfn2 abolishes insulin-induced improvements in mitochondrial function, demonstrating a causal link between mitochondrial dynamics and mechanical energy supply (Parra et al. 2014). Insulin further stimulates mitochondrial glucose oxidation independently of increased glucose uptake by activating the pyruvate dehydrogenase (PDH) complex through phosphorylation of mitochondrial Akt, GSK-3β, and PKC-δ (Karwi et al. 2020). Conversely, deletion of IR in the heart decreases mitochondrial respiration, increases oxidative stress, and impairs ATP production and fatty acid oxidation (Boudina et al. 2009). Deletion of IRS1/2 in the heart decreases ADP-stimulated mitochondrial oxygen consumption and ATP production (Riehle et al. 2013). In parallel, loss of IR signaling in cardiomyocytes compromises mitochondrial function, leading to attenuated respiratory activity, increased oxidative stress, reduced ATP synthesis, and impaired fatty acid oxidative metabolism (Boudina et al. 2009), while loss of IRS1/2 further suppresses ADP-stimulated mitochondrial respiration and limits ATP-generating capacity (Riehle et al. 2013).

Substrate utilization is tightly coupled with these processes. Insulin stimulates GLUT4 translocation via the IR–PI3K–Akt cascade (Contreras-Ferrat et al. 2014), with TBC1D4 acting as a critical phosphorylation target regulating vesicular trafficking (Achter et al. 2024). Insulin shifts myocardial substrate preference toward glucose oxidation and suppresses fatty acid β-oxidation. In CIRKO hearts, GLUT1 expression is reduced, whereas GLUT4 expression is increased, accompanied by altered expression of fatty acid oxidation enzymes (Belke et al. 2002). Taken together, insulin signaling forms a multilevel regulatory network that stabilizes cardiomyocyte mechanical homeostasis by synchronizing sarcomeric protein expression, titin-based elasticity, Ca^2^⁺ cycling dynamics, mitochondrial ATP generation, and substrate metabolism. Disruption of this network leads to altered force–velocity relationships, impaired power output, abnormal passive stiffness, energetic insufficiency, and progressive structural disintegration, ultimately culminating in mechanical failure.

### Type 2 diabetes–induced cardiomyocyte dysfunction

Type 2 diabetes alters cardiomyocyte function through coordinated changes in calcium handling, sarcomeric regulation, mechanotransduction, and metabolic control. In early phases, studies in Zucker diabetic fatty rats show increased SERCA2a mRNA expression and reduced phospholamban levels, elevating the SERCA2a/PLB ratio and enhancing sarcoplasmic reticulum calcium uptake (Fredersdorf et al. 2012). In more advanced stages, SERCA2a expression declines, contributing to impaired systolic and diastolic performance (Abe et al. 2002).

At the myofilament level, insulin-resistant cardiomyocytes exhibit increased calcium sensitivity reflected by a leftward shift in force–pCa relationships (Greenman et al. 2021; Ng et al. 2022). This effect is linked to reduced phosphorylation of cardiac troponin I at PKA-dependent sites, while total cTnI protein and O-GlcNAcylation remain unchanged (Greenman et al. 2021). Beta-adrenergic signaling is also remodeled in diabetic cardiomyocytes. In db/db mice, beta-1 receptor–mediated cAMP signaling within the SERCA2a/PLB microdomain is attenuated, whereas beta-2 signaling is enhanced, altering phospholamban phosphorylation and sarcoplasmic reticulum calcium reuptake (Lai et al. 2024). Additionally, impaired SERCA2a phosphorylation at Thr484 prolongs cytosolic calcium transients in insulin-resistant hearts (Quan et al. 2022). While rodent models show increased myofilament calcium sensitivity, human atrial trabeculae from type 2 diabetic patients exhibit reduced calcium sensitivity and elevated diastolic stress, reflecting species-specific differences and potential influences of disease stage and chamber-specific remodeling (Jones et al. 2023).

Mitochondrial dysfunction further contributes to impaired cardiomyocyte mechanics. Diabetic hearts display reduced mitochondrial respiration, impaired oxidative phosphorylation, and lower phosphocreatine/ATP ratios, indicating diminished energy availability. Insulin resistance shifts substrate utilization toward fatty acids, increasing reactive oxygen species (ROS) production and mitochondrial oxidative stress, which promotes mitochondrial damage, increased permeability transition pore opening, and disrupted coupling (Ruegsegger et al. 2018; Caturano et al. 2024). Type 2 diabetes also leads to mitochondrial fragmentation and decreased expression of fusion proteins such as MFN1, further compromising bioenergetic function (Ruegsegger et al. 2018). These alterations are expected to impair ATP-dependent calcium cycling and sarcomeric function, thereby reducing mechanical reserve under load.

Chronic inflammation, oxidative stress, and impaired protein quality control represent a unifying mechanism underlying cardiomyocyte mechanical dysfunction in type 2 diabetes. In HFpEF-DM patients, myocardial tissue exhibits elevated IL-6 and other proinflammatory markers, reduced expression of HSP27 and HSP70, and impaired mitochondrial function, collectively contributing to increased passive stiffness in isolated cardiomyocytes. Passive force can be partially restored by IL-6 inhibition, exogenous HSP administration, or treatment with the mitochondrial-targeted antioxidant mito-TEMPO, highlighting the functional impact of inflammatory and proteostatic disturbances on cardiomyocyte mechanics (Delalat et al. 2025).

Recent evidence highlights a role for the deubiquitinase OTUD1 in diabetic cardiomyopathy. OTUD1 expression is increased in type 2 diabetic cardiomyocytes, where it interacts with AMPKα2 and deubiquitinates specific lysine residues. This deubiquitination can inhibit Thr172 phosphorylation and disrupt the interaction between AMPKα2 and its upstream kinase CAMKK2, thereby suppressing AMPK activity. The resulting impairment of AMPK signaling compromises mitochondrial oxidative phosphorylation, lowers ATP availability, and exacerbates oxidative stress. Importantly, cardiomyocyte-specific OTUD1 knockout restores AMPK activation, enhances mitochondrial function, reduces reactive oxygen species, and alleviates cardiac hypertrophy and dysfunction (Han et al. 2025). Collectively, these findings indicate that type 2 diabetic cardiomyopathy arises from a complex interplay among altered calcium dynamics, sarcomeric regulation, mitochondrial bioenergetics, oxidative stress, inflammation, and impaired proteostasis, which together impair myocardial contractility and diastolic function.

### Mechanical alterations in insulin-resistant cardiomyocytes

Mechanical remodeling in insulin-resistant cardiomyocytes involves structural remodeling, increased passive stiffness, contractile dysfunction, and measurable changes in viscoelastic properties using biophysical techniques. Structural remodeling includes microtubule densification and cytoskeletal glycation driven by advanced glycation end products, thereby altering intracellular load-bearing and impairing mechanical signal transmission (Zgutka et al. 2023). Increased passive stiffness is linked to titin isoform changes and post-translational modifications that elevate titin-based tension and reduce diastolic compliance (Krüger et al. 2010a; Zhu et al. 2016; Hamdani et al. 2017). Contractile dysfunction manifests as reduced force production, impaired calcium handling, slower relaxation, and diminished contractile reserve (Janssen 2019). Viscoelastic property changes have been reported using atomic force microscopy approaches, consistent with altered elastic modulus and time-dependent mechanical behavior in diabetic cardiomyocytes (Swiatlowska and Iskratsch 2021). In this context, a synthesis of mechanical phenotypes in diabetic cardiomyocytes should integrate F_passive_, T_active_, myofilament calcium sensitivity (pCa_50_), cooperativity (nHill), rate of force redevelopment (ktr), sarcomere length–dependent activation, and stiffness/viscoelasticity metrics derived from nanoindentation and AFM (Gonçalves-Rodrigues et al 2020; Swiatlowska and Iskratsch 2021).

## Disruption of mechanotransduction in diabetes

**Fig. 2:**
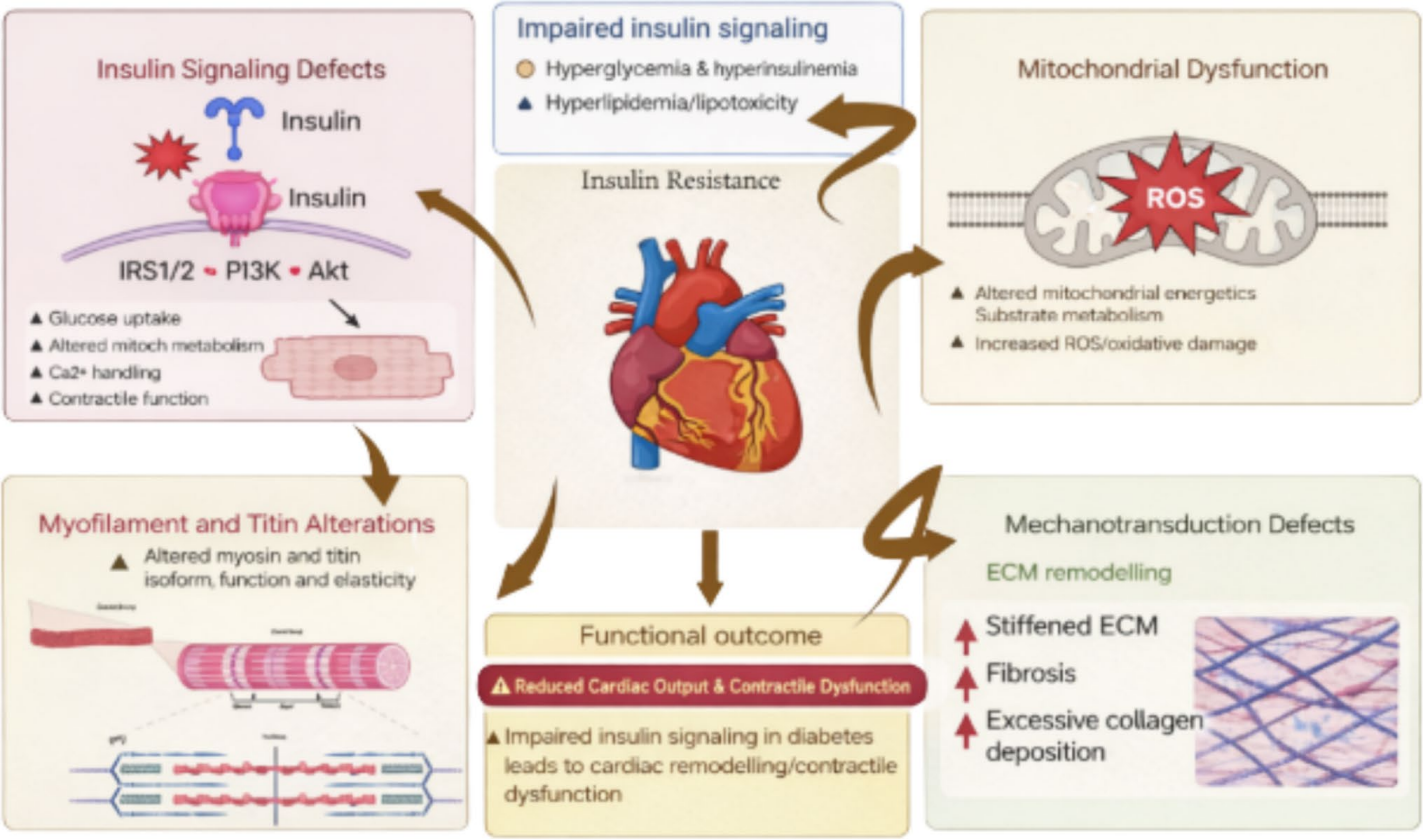
Mechanistic pathways linking insulin resistance to diabetic cardiomyopathy. Schematic showing how impaired insulin signaling (IRS1/2–PI3K–Akt disruption) in diabetes drives cardiomyocyte metabolic and calcium-handling defects, leading to reduced glucose uptake, altered mitochondrial substrate utilization, and contractile impairment. These alterations promote mitochondrial dysfunction with increased ROS production, myofilament and titin modifications that increase stiffness and impair force generation and relaxation, and extracellular matrix (ECM) remodeling characterized by fibrosis and collagen deposition. Together, these interconnected pathways converge on reduced cardiac output and contractile dysfunction characteristic of diabetic cardiomyopathy

Mechanotransduction in cardiomyocytes is an adaptive response to mechanical load that integrates excitation–contraction processes with force sensing and feedback signaling across cardiac cell types. Cardiomyocytes are continuously affected by mechanical stimuli in their microenvironment and it is a fundamental regulator of cell behavior and homeostasis (Cutroneo et al. 2012; Kowalik et al. 2026). Costameres, consisting of several proteins including integrins, play a major role in coupling the sarcomere to the sarcolemma and ECM, modulating cardiac physiology and synchronizing muscle excitation–contraction (Henderson et al. 2018). Integrins are members of the glycoprotein superfamily that contribute to cell–cell and cell–ECM interactions (Jones et al. 2015; Hein et al. 2000). They also act as mechanoreceptors and contribute to the onset of pathology when spatial organization and signaling are perturbed (Jones et al. 2015). Romanelli et al. (2024) found that diabetes mellitus induces changes in the spatial organization of costameric proteins including integrins. This spatial derangement affects mechanical and electrophysiological properties of myocardium and cardiomyocytes. In a mechanobiology context, integrin engagement and focal adhesion kinase (FAK) signaling are central to force sensing, cytoskeletal remodeling, and transcriptional adaptation, and diabetic disruption of these systems is expected to impair mechanical feedback loops that normally stabilize cellular function under chronic load (Fig. 2).

Diabetic heart disease is attributed in part to dysfunction of insulin metabolism correlated with insulin resistance, hyperinsulinemia, and hyperglycemia, leading to abnormalities in diastolic relaxation and progression toward heart failure (Marwick et al. 2018). These effects are driven by various pathways, including Yes-associated protein (YAP) and transcriptional co-activator with PDZ-binding motif (TAZ), which function as mechanotransduction effectors responding to mechanical forces and stiffness (Panciera et al. 2017). Their nuclear translocation is driven by cellular homeostasis and its disruption. Moreover, this pathway modulates cardiac development and regeneration and affects progression of cardiac remodeling and heart failure (Wang et al. 2024). Diabetes alters mechanotransduction properties of the heart and cardiomyocytes, contributing to increased cellular and left ventricular stiffness. Atomic force microscopy studies have shown that diabetic cardiomyocytes exhibit higher stiffness than nondiabetic cardiomyocytes (Romanelli et al. 2024). Alterations in morphology and structure of the diabetic heart are accompanied by changes in tissue pressure and activation of mechanical pathways, leading to specific modifications in YAP expression, nuclear translocation, and signaling (Shen et al. 2025). Mechanical stimuli, such as pressure overload, shear stress, ECM stiffness, and myocardial tension, signal to YAP/TAZ in the cytoplasm, leading to their overexpression and nuclear translocation, thereby contributing to alterations in cardiac structure and function (Shen et al. 2025). High glucose activates YAP/TAZ signaling, which promotes ECM deposition. YAP activation may enable cardiomyocyte regeneration; however, prolonged activation leads to cardiomyocyte dedifferentiation and ultimately to heart failure (Choi et al. 2023; Ikeda et al. 2019). Ultimately, diabetic cardiomyocytes respond to mechanical stress in their microenvironment, which stimulates YAP/TAZ expression and nuclear translocation, thereby altering cardiac pathophysiology and accelerating the progression of diabetic heart disease (Shen et al. 2025) (Fig. 2).

The association of diabetes and insulin resistance with cardiovascular dysfunction and heart failure is well documented (Bugger and Abel 2014). Diabetes accelerates the risk of heart failure, and heart failure itself is known as an insulin-resistant state (Cook et al. 2010; Nichols et al. 2001). Insulin signaling regulates substrate uptake and utilization, cardiac response and growth, pathological remodeling, and progression of heart failure (Abel 2021). Insulin not only regulates substrate and glucose metabolism but also augments cardiac contractility and myocardial relaxation, and because each cardiomyocyte has between 10,000 and 100,000 insulin receptors, insulin has a direct role on heart muscle via the PKB/Akt signaling pathway (Bertrand et al. 2008; Iliadis et al. 2011). Insulin increases sarcoplasmic Ca^2^⁺ influx and increases mRNA levels of ryanodine receptor and SERCA2 + -ATPase, which augment cardiomyocyte contraction and myocardial relaxation (Maier et al. 1999; Teshima et al. 2000). Insulin treatment modulates contractile response of cardiomyocytes in Langendorff and in vivo heart models via activating beta-2 adrenergic receptor–inhibitory G-protein (β2-AR–Gi) signaling, which reduces cAMP/PKA activity and consequently inhibits PKA phosphorylation of phospholamban and contractility responses (Fu et al. 2024). In a mechanobiology framework, these interactions imply that insulin resistance can impair the dynamic gain control of excitation–contraction coupling and relaxation by altering microdomain signaling and kinase access to key phosphorylation targets.

Cardiomyocytes and noncardiomyocytes are continuously exposed to mechanical and pathological stressors that drive dysfunction and remodeling in excitation–contraction coupling, stiffness, and contractile behavior; diabetes mellitus is a well-known chronic stressor. Diabetes has nanomechanical effects on cardiomyocytes leading to increased cellular and diastolic stiffness, which are hallmarks of heart failure . Cells adapt to conditions and stress factors via cell–cell junctions, ECM remodeling, and myofilament modifications. In T1DM murine models, disoriented cardiomyocyte alignment, irregular size, fragmented fibers, and interstitial collagen accumulation have been observed (Benech et al. 2014). Diminished insulin signaling can lead to abnormal ECM deposition, metabolic dysfunction, oxidative stress, and inflammation, all of which contribute to cardiomyocyte remodeling and dysfunctional contractile properties (Borghetti et al. 2018; Tan et al. 2020; Phang et al. 2023). Additionally, the diabetic heart exhibits oxidative stress due to external and internal stressors, leading to imbalanced reactive oxygen and nitrogen species (ROS and RNS) and activating NLRP3 inflammasomes, thereby initiating signaling cascades that promote cardiomyocyte apoptosis and myocardial fibrosis (Yang et al. 2018; Ritchie and Abel. 2020). Recent studies show that diabetic patients have ROS and protein kinase C activation in endothelial cells induced by hyperglycemia; this is a hallmark of diminished nitric oxide (NO) bioavailability, and similar principles apply in cardiomyocytes (Marciano et al. 2012; Sena et al. 2013; Cruz and Ryan 2017; Knapp et al. 2019). Reduced NO bioavailability is a hallmark of diastolic dysfunction, fibrosis, and altered titin phosphorylation, resulting in increased myocardial stiffness and hypertrophy (Mátyás et al. 2017).

### Biophysical tools for studying cardiomyocyte mechanics in diabetes

Excitation–contraction coupling and mechanosensitivity are key properties enabling cardiomyocytes to generate force and pump blood through the micro- and macro-circulation. Cardiomyocyte mechanics are affected by disease states and environmental factors such as ECM remodeling and fibrosis, leading to extracellular and intracellular adaptations including altered receptor–ligand interactions, force generation, stiffness, viscoelasticity, and cytoskeletal modulation (Swiatlowska and Iskratsch 2021). A variety of tools and devices are designed to measure these adaptations and modulations, and it is useful to link each technique to the physical parameter quantified and the spatial/temporal scale of measurement.

Atomic force microscopy (AFM) is a widely used technique for studying cardiac mechanobiology by measuring nanomechanical properties such as stiffness and viscoelasticity. AFM can also measure force-dependent domain folding and unfolding events when operated in single-molecule mode, either by molecules attached to a cantilever tip or by using magnetic or optical tweezers. These systems apply force through a piezo-controlled cantilever, a magnetic field (magnetic tweezers), or a laser beam–based dielectric bead (optical tweezers) (Neuman and Nagy 2008; Swiatlowska and Iskratsch 2021). In diabetes, AFM-based measurements showing increased cardiomyocyte stiffness (Romanelli et al. 2024) provide direct evidence of altered elastic properties at the single-cell level and can be integrated with titin-based and cytoskeletal mechanisms.

Traction force microscopy (TFM) can be used to monitor correlation of contractile structure and function of isolated cardiomyocytes by quantifying planar substrate displacement under adherent cells (Pasqualini et al. 2018; Aratyn-Schaus et al. 2016; Schoen et al. 2010). However, the sensitivity of this technique is influenced by substrate mechanics, ECM composition, and microscope properties (Pasqualini et al. 2018). In diabetic remodeling, where ECM stiffness and adhesion signaling are altered, careful calibration and interpretation are essential to avoid conflating cellular force defects with substrate-dependent readouts.

Aurora Scientific 600 A systems measure force, stiffness, rate of force redevelopment (ktr), and Ca^2^⁺ sensitivity in skinned or permeabilized cardiomyocytes. The system includes a piezo motor to stretch cardiomyocytes and a force transducer to measure isometric contraction. The method is based on a skinned cardiomyocyte glued between two needles (force and motor), stretched from one side, and exposed to defined Ca^2^⁺ solutions (pCa 4.5–9) to quantify stiffness and Ca^2^⁺ sensitivity. Force is normalized to cross-sectional area to obtain active tension (T_active_, kN·m^−2^), passive tension (F_passive_, kN·m^−2^), myofilament Ca^2^⁺ sensitivity (pCa50), myofilament cooperativity (nHill), the rate of force redevelopment (ktr), and sarcomere length dependence of these parameters (Gonçalves-Rodrigues et al. 2020). This approach is particularly relevant for diabetes because it isolates myofilament-level mechanical effects from upstream calcium handling, allowing direct measurement of altered pCa50 and ktr consistent with changes in troponin phosphorylation and sarcomeric regulation (PMID: 34605091; PMID: 36207382).

IonOptix systems are designed to measure intracellular Ca^2^⁺ and mechanical properties, including contractility, work loops, and sarcomere length, in single cardiomyocytes, iPSC-derived cardiomyocytes, and cardiac tissue in real time. The system can simultaneously detect Ca^2^⁺ transients, contractility, and action potential properties using fluorescent dyes such as fura-2, enabling correlation between signaling and motion. The platform is commonly used for studying heart failure, arrhythmia (including using MyoPacer EP), atrial fibrillation, and other cardiovascular and muscular diseases. Additional modules (MultiCell High-Throughput System, CytoMotion Lite, MyoStretcher, MyoClamp) support non-fluorescent contractility, force measurements in slices, and controlled stretch paradigms. In the context of diabetes, IonOptix-like approaches are well suited to quantify prolonged calcium transients (Al Kury et al. 2021), altered relaxation kinetics, and the coupling between energetic stress and mechanical output.

In addition to the techniques above, numerous tools exist to measure cardiac and cardiomyocyte properties, including video-based motion analysis, electrophysiological approaches (patch clamp, action potential, and field potential recording), microfluidic devices, microscale tools, nanotubes, nanofibers, and mechano-scanning ion conductance microscopy (mechano-SICM), among others. These approaches can be linked to specific readouts including membrane excitability, ionic current density, force generation, stiffness, and viscoelastic parameters, and they enable multimodal integration in which electrical and mechanical phenotypes are quantified simultaneously.

### Therapeutic perspectives and future directions

To treat diabetic heart disease, several classes of therapies are used to manage symptoms and improve outcomes, including sodium-glucose cotransporter 2 (SGLT2) inhibitors, glucagon-like peptide 1 (GLP-1) agonists, sulfonylureas, and dipeptidyl peptidase-4 (DPP-4) inhibitors. These agents modulate glycemic levels, reduce body mass, lower oxidative stress, and reduce blood pressure (Tan et al. 2020; Margulies et al. 2016; Castellana et al. 2019). From a mechanobiology perspective, the most relevant question is how these therapies alter mechanical properties such as titin-based stiffness, myofilament phosphorylation, calcium cycling efficiency, and energetic reserve rather than glucose alone.

SGLT2 inhibitors reduce renal glucose reabsorption by blocking SGLT2 cotransporters in proximal tubules and are among the most promising therapies for diabetic cardiomyopathy and HF, particularly HFpEF (Anker et al. 2021; Phang et al. 2023; Fonseca Correa and Correa Rotter 2021). Numerous studies show that SGLT2 inhibitors have cardioprotective roles via preventing cardiac remodeling, ameliorating diastolic function and contractility, improving Ca^2^⁺ and Na⁺ homeostasis, and reducing oxidative stress and inflammation in heart failure patients with diabetes (Pabel et al. 2021; Phang et al. 2023). Pabel et al. (2018) reported that empagliflozin significantly reduces passive stiffness by increasing phosphorylation of myofilament proteins via the NO–sGC–cGMP–PKG signaling pathway in patients with HFpEF.

GLP-1 was primarily designed to treat T2DM patients; however, recent studies show that it also reduces cardiovascular risk (Drucker 2016; Yang 2025). GLP-1 induces the PI3K/Akt signaling cascade known as an insulin-independent cardioprotective signaling pathway (Marx et al. 2022; Zhu et al. 2023; Timmers et al. 2009). Treating HFpEF models with GLP-1 agonists leads to improved cardiac ATP production and heart contractility (Kosiborod et al. 2024). GLP-1 also reduces cardiac remodeling and myocardial collagen levels, improving diastolic function (Zhu et al. 2023). Taken together, both drug classes can influence kinase signaling pathways that modulate myofilament phosphorylation, stiffness, and excitation–contraction coupling, and these effects can be quantified using the mechanical tools discussed above.

The diabetic drug metformin, commonly used for T2DM patients, improves LV diastolic function in patients, titin N2B isoform knockout (KO) mice, and HFpEF-like characteristics transverse aortic constriction (TAC) mice (Slater et al. 2019). Additionally, the authors revealed that metformin supplementation increases phosphorylation of PKA sites in titin N2B isoform, but not in PEVK sites which are related to improved titin elasticity and diastolic function. Overall, the study revealed that metformin ameliorates diastolic LV stiffness, titin-based stiffness, exercise capacity, and increase in titin compliance.

Targeting mechanical pathways directly is an emerging therapeutic theme. Diabetes is a driver of numerous alterations in mechanical properties of the heart, including contractility, excitation–contraction uncoupling, stiffness, systolic and diastolic dysfunction, and myofilament proteins modifications. The giant spring protein titin is a major modulator of diastolic function and mechanical properties of the heart via governing myocardial stiffness and elasticity. Alterations in isoform ratio and post-translational modifications, especially phosphorylation, have crucial roles in stiffness and elasticity, and in the diabetic heart, this is a well-known phenomenon (Linke and Hamdani 2014; Herwig et al. 2025). Post-translational modifications of titin via kinases such as PKG/A/C and CaMKII are established drivers of F_passive_, while the effect on passive stiffness depends on which region of titin is phosphorylated (Hamdani et al. 2013; Herwig et al. 2024; Krüger et al. 2009). In our study, PKG- and PKA-dependent reduced titin phosphorylation at different N2Bus sites was higher in the diabetic heart compared to the nondiabetic heart, and treating cardiomyocytes with PKG and PKA reduced F_passive_ (Herwig et al. 2025). Fibrosis and ECM remodeling also modulate F_passive_, and patients with diabetes often have higher fibrosis and advanced glycation end product deposition (Berg et al. 1999). Higher LV stiffness in HF patients with diabetes is related to fibrosis and AGEs compared to nondiabetic HF patients (van Heerebeek et al. 2008). Impaired cGMP/PKG and PKCα activity, LV diastolic dysfunction, AGE deposition, ECM remodeling, and titin isoform switching are common phenotypes in diabetic HF patients (Hopf et al. 2018; Falcão-Pires et al. 2011). AGEs induce inflammation and impair insulin signaling and NO levels, kinase activity, and fibrosis (Delbridge et al. 2016; Jia et al. 2018). These pathways define clear mechanistic targets that can be evaluated by measuring cardiomyocyte stiffness, passive tension, and viscoelastic behavior before and after intervention.

Emerging models, including iPSC-derived cardiomyocytes and engineered tissues, provide new opportunities to study diabetic mechanics and mechanotransduction. Given the duration, complexity, and limitations of patient-based research, developing new models and methods is an urgent need for drug screening and disease modeling. Even though it is challenging to obtain induced pluripotent stem cell-derived cardiomyocytes (iPSC-CMs) with adult-like maturation, their functionality and structure can approximate key properties of cardiomyocytes, and they have promising potential to study and mimic cardiovascular diseases and comorbidities such as diabetes. Carson et al. (2016) plated iPSC-CMs on nanogrooved topographies to mimic ECM fiber orientation and showed successful functionality with arginylglycylaspartic acid (RGD) for cell adhesion. Lin et al. (2014) used randomly oriented electrospun patches to recapitulate ECM structure and plated cardiomyocytes on aligned substrates, observing improved beating. When these patches were applied to infarcted hearts, in vivo performance improved with ameliorated hemodynamic parameters, echocardiography, reduced infarct size, and aligned anisotropic cell morphology. Similarly, nanofiber scaffolds that mimic ECM organization improved cardiac maturation, produced elongated nuclei, and synchronized Ca^2^⁺ fluctuations in iPSC-derived cardiac cells (Ding et al. 2020). These approaches emphasize that mechanics and microenvironmental architecture can drive maturation and functional phenotype, a central theme in mechanobiology in diabetes.

Microfluidics (heart-on-chip) provides a proven approach to develop and investigate in vitro cell models derived from microanalytical techniques such as gas chromatography and capillary electrophoresis (Whitesides 2006; Wang et al. 2025). The method supports heterogeneous cell cultures and biomimetic in vivo-like environments for high-throughput drug screening and disease modeling (Cui and Wang 2019). Microfluidic systems are effective for modeling cardiac diseases such as hypertrophy, atrial fibrillation, arrhythmia, stroke, and heart failure structure and function. They can replicate extracellular microenvironment conditions and deliver mechanical, chemical, and electrical stimuli while enabling electromechanical and electrophysiological measurements, contractile function readouts, Ca^2^⁺ metabolism, and excitation–contraction coupling assessments ( Bhatia and Ingber 2014; McCain et al. 2013; Huang and Lei 2023).

Engineered heart tissue (EHT) is a miniaturized heart-based approach enabling evaluation of contractile function (Hansen et al. 2010). The technique typically uses cardiac muscle tissues attached to flexible pillars; video-based analysis of pillar deflection yields force, contraction frequency, contraction–relaxation times, and systolic/diastolic behavior (Wang et al. 2025). EHT provides an advanced platform to investigate drug efficacy, cardiac mechanics, and disease modeling in a controlled mechanical context. Overall, these techniques enable deep investigation of diabetic cardiac mechanics and mechanotransduction. Overall, these platforms enable mechanistically grounded testing of diabetic cardiomyocyte mechanics and mechanotransduction under controlled biochemical and mechanical boundary conditions. Interpretation of mechanical measurements requires careful control of loading conditions, substrate stiffness, temperature, pacing frequency, and species/model differences. In particular, changes in stiffness measured by AFM or indentation can reflect titin, cytoskeleton, and ECM contributions depending on preparation, while force and Ca^2^⁺ sensitivity measured in skinned cardiomyocytes isolate myofilament properties but remove upstream signaling.

## Conclusion and outlook

Insulin signaling stabilizes cardiomyocyte mechanical homeostasis by coordinating myofilament protein composition and phosphorylation, titin splicing and phosphorylation, calcium handling and microdomain signaling, mitochondrial dynamics and ATP production, and substrate metabolism. Type 2 diabetes disrupts this integrated network and produces a composite mechanical phenotype characterized by altered force–pCa relationships, impaired contractile kinetics, increased passive stiffness, modified viscoelastic behavior, energetic insufficiency, and defective mechanotransduction involving costameres, integrins/FAK, and YAP/TAZ signaling. These alterations are not only molecular but quantifiable physical changes measurable through AFM, TFM, skinned-cell mechanics, real-time calcium–motion platforms, EHT systems, and microphysiological heart-on-chip devices. Future work will benefit from integrating multiscale mechanical measurements with signaling and proteostasis readouts, testing therapies that directly restore mechanical pathways such as NO–cGMP–PKG–titin phosphorylation and AGE reduction and leveraging iPSC/EHT/microfluidic models to systematically map causal links from insulin resistance to mechanical failure.

## Data Availability

No datasets were generated or analyzed during the current study.
